# Molecular Insights into the Inhibition of Lipid Accumulation in Hepatocytes by Unique Extracts of Ashwagandha

**DOI:** 10.3390/ijms252212256

**Published:** 2024-11-14

**Authors:** Dongyang Li, Hanlin Han, Yixin Sun, Huayue Zhang, Ren Yoshitomi, Sunil C. Kaul, Renu Wadhwa

**Affiliations:** 1 AIST-INDIA DAILAB, National Institute of Advanced Industrial Science and Technology (AIST), Tsukuba 305-8565, Japans2130297@u.tsukuba.ac.jp (H.Z.); s-kaul@aist.go.jp (S.C.K.); 2 Graduate School of Science and Technology, University of Tsukuba, 1-1-1 Tennodai, Tsukuba 305-8577, Japan

**Keywords:** steatosis, stress, CARF expression, Ashwagandha, withanolides, lipogenesis, regulation, treatment

## Abstract

We investigated the effect of purified withanolides and extracts derived from Ashwagandha on steatosis, the abnormal accumulation of fat that can lead to non-alcoholic fatty liver disease (NAFLD). Collaborator of ARF (CARF, also known as CDKN2AIP, a protein that regulates hepatic lipid metabolism, fat buildup, and liver damage) was used as an indicator. Six withanolides (Withaferin A, Withanone, Withanolide B, Withanoside IV, Withanoside V, and Withanostraminolide-12 deoxy) reversed the decrease in CARF caused by exposure to free fatty acids (FFAs) in liver-derived cells (HepG2 hepatocytes). After analyzing the effects of these withanolides on CARF mRNA and protein levels, FFA accumulation, protein aggregation, and oxidative and DNA damage stresses, we selected Withaferin A and Withanone for molecular analyses. Using the palmitic-acid-induced fatty acid accumulation stress model in Huh7 cells, we found a significant reduction in the activity of the key regulators of lipogenesis pathways, including sterol regulatory element-binding protein-1c (SREBP-1c), fatty acid synthase (FASN), and peroxisome proliferator-activated receptors (PPARγ and PPARα). This in vitro study suggests that low, non-toxic doses of Withaferin A, Withanone, or Ashwagandha extracts containing these withanolides possess anti-steatosis and antioxidative-stress properties. Further in vivo and clinical studies are required to investigate the therapeutic potential of these Ashwagandha-derived bioactive ingredients for NAFLD.

## 1. Introduction

Ashwagandha (*Withania somnifera*) is an ayurvedic herb commonly used for various health benefits in the Indian traditional home-medicine system. It is enriched in steroidal lactones (withanolides), the active ingredients for various bioactivities. Among these, Withaferin A (Wi-A) and Withanone (Wi-N) have been shown to possess good anticancer activity that works through multiple mechanisms [1,2,3,4]. Among different cancers, it is more effective against breast cancer, followed by colon, lung, prostate, and blood cancer [5,6]. Furthermore, low concentrations of these withanolides have been suggested to protect cells against various stresses connected to their antiaging potential, including improved memory and brain function, as supported by in vitro and in vivo studies [3,7,8,9,10,11].

Several studies have reported the clinical efficacy of Ashwagandha extracts for the regulation of body fat. Reductions in body weight as well as total and LDL cholesterol were reported in a study of healthy volunteers [12]. In a study of high-fat-diet (HFD)-induced obese mice, the dietary administration of Wi-A (1.25 mg/kg/day) reduced hepatic inflammation, oxidative stress, and insulin resistance in the mice. The HFD group showed reduced serum pro-inflammatory cytokines and hepatic mRNA levels of *TLR4*, *NFκB*, *TNF-α*, and *COX2*. Wi-A resulted in significant improvements in hepatic antioxidative enzyme activities, insulin sensitivity, adipocytokines, and enhanced lipid and glucose metabolism that were mediated by an increase in hepatic peroxisome proliferator-activated receptor (PPAR)γ, CD36 (also known as fatty acid translocase), and flavin adenine dinucleotide (FAD) mRNA [13]. Xia et al. [14,15] reported the hepatoprotective function of Wi-A in mice models of fulminant hepatitis (FH, an incurable clinical syndrome). A significant mitigation of GalN/LPS-induced hepatotoxicity was shown to be mediated by macrophage and NLRP3 antagonism targeting, suggesting Wi-A to be a candidate drug for the treatment of FH, liver diseases, and cancer [14,15]. Another study on non-alcoholic steatohepatitis (NASH, the progressive stage of non-alcoholic fatty liver disease that highly increases the risk of cirrhosis and liver cancer) used two well-defined mice models, namely, a methionine-choline-deficient diet and an HFD. Biochemical and histological analyses on the control and Wi-A-treated groups showed preventive and therapeutical effects on liver injury, as marked by lower serum aminotransaminases, hepatic steatosis, liver inflammation, and fibrosis in both models [16]. Ashwagandha has been shown to possess the ability to lower blood glucose levels. Wi-A was shown to control type 1 diabetes induced in rats through the modulation of Nrf2/NFκB signaling [17]. A study on a hypercholesterolemia rat model showed reduced cholesterol levels in the *Withania somnifera* group [18]. Clinical studies have reported improved lipidemic profiles, body weight, and blood pressure [19,20,21]. 

Non-alcoholic fatty liver disease (NAFLD), represented by the accumulation of fatty acids in hepatocytes, is an early stage of NASH and is closely connected to metabolic dysfunctions such as insulin resistance, dyslipidemia, and cardiovascular disease [22]. Molecular studies have defined the key role of transcription factor PPARγ in lipogenesis [23]. Hepatocytes treated with free fatty acids (FFAs) show an upregulation of PPARγ that controls fatty acid uptake and de novo lipogenesis mediated through sterol regulatory element-binding protein-1c (SREBP-1c) [23,24]. An overload of FFAs induces mitochondria dysfunction, endoplasmic reticulum (ER) stress, and oxidative stress that, in turn, exacerbate lipid accumulation and liver damage by disrupting normal lipid metabolism [25]. In consensus with this, the attenuation of ER stress by phytochemicals has been suggested to be a therapeutic approach for NAFLD and NASH [25]. Sirtuin 2, a nicotinamide adenine dinucleotide NAD(+)-dependent deacetylase, has been shown to alleviate ER and oxidative stress and promote liver regeneration, and has been suggested to be a therapeutic target for NAFLD and NASH [26]. Sirtuin 1 deficiency is also associated with NAFLD; its overexpression has been shown to alleviate NAFLD by enhancing mitochondrial function, suppressing oxidative stress, and reducing apoptosis and inflammation [27]. 

Recent studies have highlighted the role of the collaborator of ARF (CARF, also known as CDKN2AIP, a stress-regulating protein that controls the proliferative fate of cells from apoptosis and growth arrest to malignant transformation) in regulating hepatic lipid metabolism [28,29,30,31,32,33]. CARF expression is suppressed by fatty acid overloads, leading to increased ER stress and reactive oxygen species (ROS) accumulation, exacerbating lipid accumulation and liver damage. The exogenous expression of CARF was seen to protect against NAFLD, reducing ER stress and ROS levels in in vitro and in vivo studies [33]. RNA sequencing analyses following CARF silencing in HepG2 cells revealed alterations in the genes regulating lipogenesis and β-oxidation, underscoring CARF’s essential role in lipid regulation. These studies suggest that modulation of the CARF expression is a promising approach to alleviate ER stress and oxidative damage, thereby improving liver health and preventing the progression of NAFLD [33]. Thus, understanding the interplay between ER stress, ROS accumulation, and CARF expression seems to be crucial for the development of new treatment modalities for NAFLD. In the present study, we validated the association between CARF expression and FFA accumulation in hepatocytes, identified withanolides and extracts from Ashwagandha that inhibited this process, and determined their molecular mechanism of action. 

## 2. Results

### 2.1. Downregulation of Collaborator of ARF (CARF) by Free Fatty Acid (FFA)-Induced Lipid Stress and Its Recovery via Ashwagandha Withanolides

**Figure 1:**
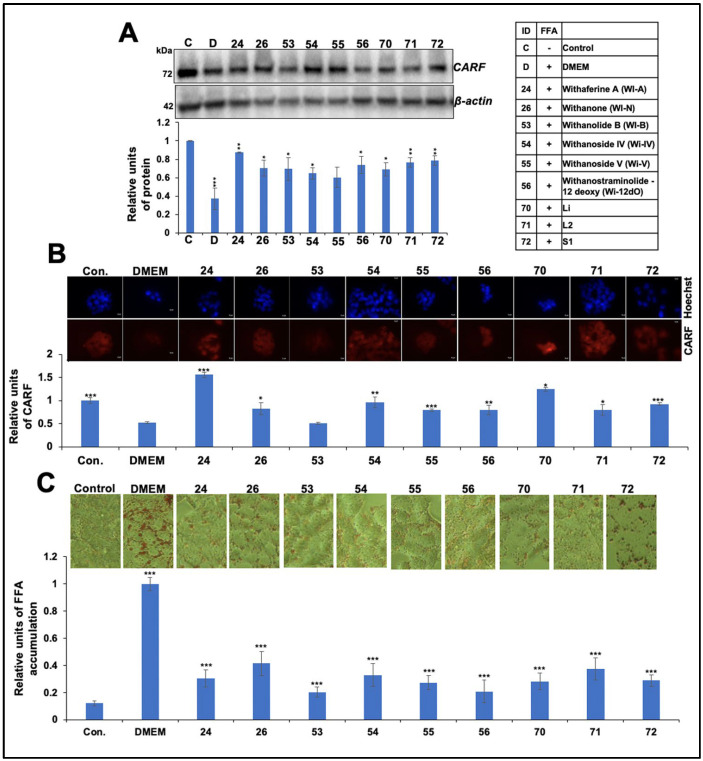
Protective effects of Ashwagandha withanolides and extracts on collaborator ARF CARF expression and lipid accumulation in free fatty acid (FFA)-treated HepG2 cells. (**A**) Western blot analysis showing a decrease in CARF expression in HepG2 cells treated with FFAs and its reversal in cells co-treated with Ashwagandha withanolides and extracts. β-actin was used as an internal loading control. Quantitation from three independent experiments is shown below. (**B**) Immunostaining of CARF showing a remarkable decrease in CARF in cells treated with FFAs and its reversal in cells co-treated with Ashwagandha withanolides and extracts. Quantitation from three independent experiments is shown below. (**C**) Oil Red O staining showing an increase in lipid accumulation in FFA-treated HepG2 cells and its reversal in cells co-treated with Ashwagandha withanolides and extracts. Quantification from three independent experiments is shown below. The quantification graph data in (**A**–**C**) were normalized against the FFA group and plotted as fold differences (mean ± SD; *n* = 3). * *p* < 0.05, ** *p* < 0.01, and *** *p* < 0.001 denote the statistical significance compared with the FFA group (derived from an unpaired Student’s *t*-test). (**B**,**C**) images were captured under a magnification of 20×.

We initially treated HepG2 cells with FFAs and determined their effect on cell viability. As shown in Supplementary Figure S1A, FFAs concentrations from 0.125 to 1.0 mM did not cause cytotoxicity. Cells treated with these concentrations showed a dose-dependent increase in lipid accumulation (Supplementary Figure S1B). Of note, the increase in cellular lipid content was marked by a decrease in CARF, as determined by Western blotting and immunostaining (Supplementary Figure S1C–E). Next, we determined the non-toxic concentrations of 6 withanolides and their mixture in leaf and stem extracts, as described in the Section 4. As shown in Supplementary Figure S2A, HepG2 cells treated with Wi-A (0.1 μM), Wi-N, Wi-B, Wi-IV, Wi-V, or Wi-12dO (each 5 μM) did not show cytotoxicity; the change in viability was less than 10–15%. The cells treated with a water-based extract from leaves (L1) and stems (S1) showed a 23% and 18% decrease in viability, respectively. The DMSO-based extract from leaves (L2) did not show any effect. Next, we recovered the FFA-treated cells in a withanolide-supplemented medium and examined their CARF expression level. Figure 1A,B show that the FFA-treated cells that were recovered in a normal culture medium showed decreased CARF protein levels, as determined by Western blotting and immunostaining.

The recovery of FFA-stressed cells in a medium supplemented with most of the withanolides caused the recovery of the CARF expression. Maximum recovery of the CARF expression in FFAs and withanolide-treated cells was seen in the Wi-A (#24)-supplemented medium. As predicted by changes in the CARF expression, lipid accumulation increased in the stressed cells. Notably, the cells recovered in the withanolide-supplemented DMEM showed a remarkable reduction in lipid accumulation compared with those recovered in the control DMEM (Figure 1C). 

### 2.2. Ashwagandha Withanolides Protect Cells Against Oxidative and Metal Stress

**Figure 2:**
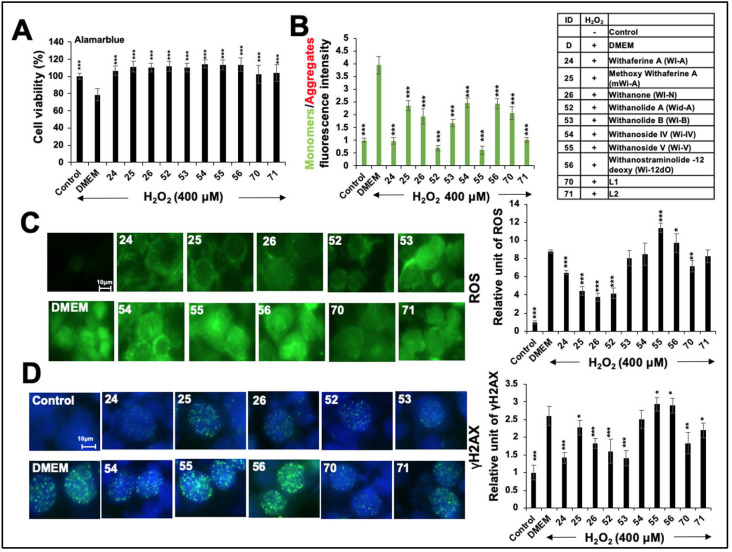
Protective effects of Ashwagandha withanolides and extracts on H_2_O_2_-induced oxidative stress in HepG2 cells. (**A**) Cell viability assay indicating that Ashwagandha withanolides and extracts protected HepG2 cells against a decrease in viability caused by a 400 μM H_2_O_2_ treatment. (**B**) JC-1 fluorescence intensity, plotted as a ratio of monomers (green, indicative of depolarized membrane potential) to aggregates (red, indicative of intact mitochondrial membrane potential), showing that Ashwagandha withanolides and extracts protected HepG2 cells from H_2_O_2_-induced membrane depolarization. (**C**) Representative images of intracellular reactive oxygen species (ROS) levels in HepG2 cells under oxidative stress induced by H_2_O_2_, demonstrating the protective effects of some withanolides and Ashwagandha extracts. Quantitation from three independent experiments is shown on the right. (**D**) Immunostaining of γH_2_AX foci indicating the protective effects of some withanolides and Ashwagandha extracts on DNA damage in HepG2 cells treated with H_2_O_2_. Quantification from three independent experiments is shown on the right. For panels (**A**,**B**) and the quantification graphs in (**C**,**D**), data were normalized against the stress group and plotted as fold differences. The statistical significance of the data (mean ± SD; *n* = 3; and * *p* < 0.05, ** *p* < 0.01, and *** *p* < 0.001) was calculated using an unpaired Student’s *t*-test.

Based on the established role of oxidative stress in lipid-accumulation-related liver dysfunctions, we next determined the effect of withanolides on oxidative stress. Methoxy-withaferin A (mWi-A) and Withanolide A (Wid-A) have previously been shown to possess antistress properties [6,10,34] and, hence, were included along with the other withanolides. Cells treated with H_2_O_2_ at a concentration that compromised their viability by ~25% (Supplementary Figure S2B) were recovered in the withanolide-supplemented medium. As shown in Figure 2A, cells recovered in each withanolide and extract (#70-L1 and #71-L2)-supplemented medium showed higher recovery than those recovered in DMEM. We examined mitochondrial depolarization and dysfunction in the stressed and recovered cells. As shown in Figure 2B, quantitation of the data revealed a 4-fold increase in the depolarization of the mitochondrial membrane, as revealed by an increased JC-1 monomer (green)/aggregate (red) ratio in oxidatively stressed cells. On the other hand, cells recovered in the withanolide-supplemented medium showed a decrease in the JC-1 ratio compared with the control stressed cells. A remarkable reduction in the JC-1 ratio was found in the cells recovered in the medium supplemented with Wi-A, Wid-A, Wi-V, or #71-L2.

Live imaging of ROS exhibited intense staining in the oxidatively stressed cells recovered in DMEM. Cells recovered in a medium supplemented with most of the withanolides showed a maximum ROS diminution in Wi-N, followed by mWi-A-, Wid-A-, and Wi-A-treated cells (Figure 2C). On the other hand, cells recovered in Wi-B/Wi-IV did not show any difference, and the cells recovered in Wi-V/Wi-12dO showed an increase in ROS. Among the two extracts, #70-L1 caused a small but significant decrease in oxidative-stress-induced ROS, but #71-L2 was effective. We also investigated DNA damage in the control, stressed, and recovered cells. As shown in Figure 2D, there was a 2.5-fold increase in γH2AX foci in the oxidatively stressed cells. Cells recovered in several withanolides (including Wi-A, mWi-A, Wi-N, Wid-A, and Wid-B) showed reduced γH2AX foci. Notably, and consistent with the data on ROS accumulation, cells recovered in Wi-V/Wi-12dO showed an increase in DNA damage. On the other hand, cells recovered in either #70-L1 or #71-L2-supplemented mediums showed an attenuation of the oxidative-stress-induced increase in γH2AX foci.

**Figure 3:**
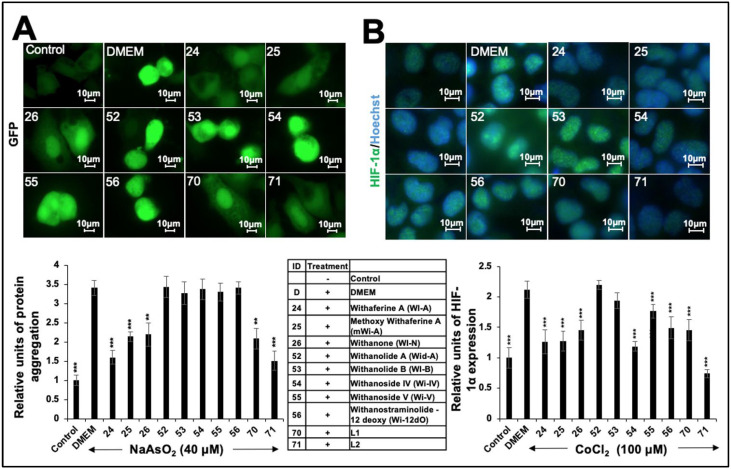
Effects of Ashwagandha withanolides and extracts on protein aggregation and hypoxia stress. (**A**) GFP-expressing HepG2 cells treated with NaAsO_2_ showed aggregated/increased GFP intensity. Cells treated with Ashwagandha withanolides and extracts exhibited a reduction in GFP aggregates. Quantification from three independent experiments is shown below. (**B**) Immunostaining for HIF-1α in HepG2 cells treated with CoCl_2_, showing an increase. Cells treated with Ashwagandha withanolides and extracts showed a reduction in HIF-1α. Quantitation from three independent experiments is shown below. For the quantitation graphs in (**A**,**B**), data were normalized against the stressed groups and plotted as fold differences. The statistical significance of the data (mean ± SD; *n* = 3; and ** *p* < 0.01, and *** *p* < 0.001) was calculated using an unpaired Student’s *t*-test.

We also determined the effect of these withanolides on protein aggregation stress induced by the treatment of cells with heavy metals. GFP was used as a reporter. The cells (stably transfected with GFP) were treated with NaAsO_2_ (40 μM) and recovered in a normal medium that showed GFP aggregation (Figure 3A). Notably, cells recovered in Wi-A-, mWi-A-, Wi-N-, #70-L1-, or #71-L2-supplemented mediums showed a remarkable reduction in protein aggregation (Figure 3A). As expected, cells challenged with CoCl_2_ (100 μM) showed an upregulation of hypoxia signaling (Figure 3B). Recovery in withanolide (Wi-A, mWi-A, Wi-N, or Wi-IV)- or extract (#70-L1 or #71-L2)-supplemented mediums caused the remarkable downregulation of metal-induced hypoxia signaling (Figure 3B). Combining the above data on the effect of withanolides/extracts on stressed cells, we selected Wi-A, Wi-N, and the extracts (represented a complex mixture of withanolides) for further molecular analyses on lipogenesis signaling.

### 2.3. Molecular Mechanism of Protection Against Lipid Stress by Withanolides

We next sought to determine the molecular mechanism(s) of the effect of withanolides on stress and lipid accumulation. Huh7 cells, which have previously been shown to possess higher intracellular triglyceride (TG) capacity, liver-specific differentiation, and metabolic pathways than HepG2 cells [35,36,37,38], were selected. Furthermore, these cells have been shown to activate ER stress, inflammation, and lipid accumulation pathways in response to a saturated fatty acid (palmitic acid (PA)) [39,40,41]. A cell viability assay showed a dose-dependent reduction in viability in PA-treated Huh7 cells; 100 μM PA was selected as a non-toxic dose (Supplementary Figure S3A). The increase in intracellular lipid accumulation in cells treated with non-toxic concentrations of PA was confirmed by Oil Red O staining and quantified using a TG detection kit (Supplementary Figure S3B,C). Furthermore, cells treated with leaf (L; high amount of total withanolides) and stem (S; low amount of total withanolides) extracts with a high ratio of Wi-A (1) or Wi-N (2) showed the attenuation of a PA-induced increase in lipid accumulation (Supplementary Figure S3D,E). As Wi-A and Wi-N are the main bioactive compounds in Ashwagandha leaf and stem extracts [42], we next subjected PA-treated Huh7 to recovery in a medium supplemented with non-toxic concentrations of either Wi-A (0.1 μM) or Wi-N (5 μM).

**Figure 4:**
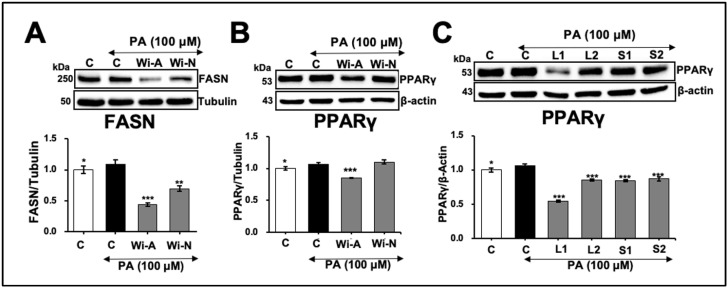
Effects of withanolides and Ashwagandha extracts on key regulators of lipid metabolism. (**A**) Western blot showing the effect of Withaferin A (Wi-A) and Withanone (Wi-N) on fatty acid synthase (FASN) protein level in Huh7 cells treated with PA. Quantitation from three independent experiments is shown below. (**B**) Western blot displaying the effect of Wi-A and Wi-N on peroxisome proliferator-activated receptor (PPAR)γ protein levels in palmitic acid (PA)-treated Huh7 cells. Quantitation from three independent experiments is shown below. (**C**) Western blot analysis showing the effect of Ashwagandha extracts on PPARγ protein levels in Huh7 cells treated with PA. Quantitation from three independent experiments is shown below. For the quantitation graphs, data were normalized against the PA group and plotted as fold differences (mean ± SD; *n* = 3). The statistical significance of the data (mean ± SD; *n* = 3; and * *p* < 0.05, ** *p* < 0.01, and *** *p* < 0.001) was calculated using an unpaired Student’s *t*-test.

Figure 4A shows that FASN and PPARγ, the key regulators of hepatic lipid accumulation, increased in PA-treated cells recovered in a normal medium. On the other hand, the cells recovered in either Wi-A- or Wi-N-supplemented mediums showed a significant decrease in both proteins (Figure 4A,B). Notably, Wi-A caused a more substantial effect than Wi-N for both target proteins that were further validated in cells recovered in a medium supplemented with leaf and stem extracts. As shown in Figure 4C, the L1 extract, possessing a high content of Wi-A, showed a substantial reduction in the PPARγ expression.

**Figure 5:**
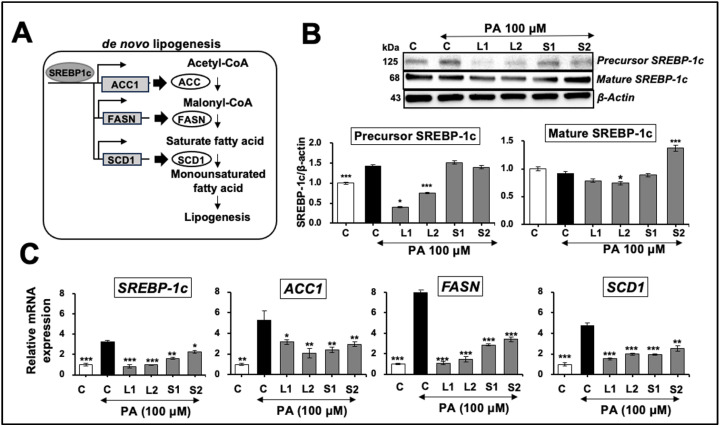
Effects of Ashwagandha extracts on the de novo lipogenesis pathway. (**A**) A schematic representation of the de novo lipogenesis pathway, key enzymes, and steps involved in fatty acid synthesis is shown. (**B**) Western blot showing the effects of Ashwagandha extracts on precursor and mature sterol regulatory element-binding protein-1c (SREBP-1c) expression levels in Huh7 cells treated with PA. β-actin was used as a loading control. Quantitation from three independent experiments is shown below. (**C**) Ashwagandha extracts decreased *SREBP-1c* mRNA and its target genes Acetyl-CoA Carboxylase 1 (*ACC1*), fatty acid synthase (*FASN*) and stearoyl-CoA desaturase 1 (*SCD1*) in PA-treated HepG2 cells. Data were normalized against the PA group for all panels and plotted as fold differences (mean ± SD; *n* = 3). The statistical significance of the data (mean ± SD; *n* = 3; and * *p* < 0.05, ** *p* < 0.01, and *** *p* < 0.001) was calculated using an unpaired Student’s *t*-test.

De novo lipogenesis: Based on the above data, non-toxic concentrations of Ashwagandha extracts rich in either Wi-A or Wi-N [42] were selected for further molecular analyses. Cells were pretreated with non-toxic concentrations of L1, L2 (0.05%), S1, or S2 (0.1%) extracts for 24 h, followed by 100 μM PA for 24 h. Western blotting for SREBP-1, a key regulator of de novo lipogenesis (Figure 5A), showed increased precursor, but not mature, SREBP-1c in PA-treated cells (Figure 5B). Of note, L1- and L2-pretreated cells showed a remarkable reduction in precursor SREBP-1c; S1- and S2-treated cells did not show such an effect on SREBP-1c precursor protein (Figure 5B). As SREBP-1c upregulates the expression of several genes in the FA biosynthetic pathway (Figure 5A) [43], we next examined the mRNA expression of *SREBP-1c* and its effector genes, Acetyl-CoA carboxylase 1 (*ACC1*), FASN, and stearoyl-CoA desaturase 1 (*SCD1*). As shown in Figure 5C, *SREBP-1c*, *ACC1*, *FASN*, and *SCD1* mRNAs were upregulated in PA-treated cells compared with the controls. Cells pretreated with L1, L2, S1, and S2 extracts showed a downregulation (Figure 5C). L1- and L2-treated cells showed a stronger reduction in the mRNA expression of all four genes; the maximum was in *FASN*, followed by *SREBP-1c*, *SCD1*, and *ACC1*.

**Figure 6:**
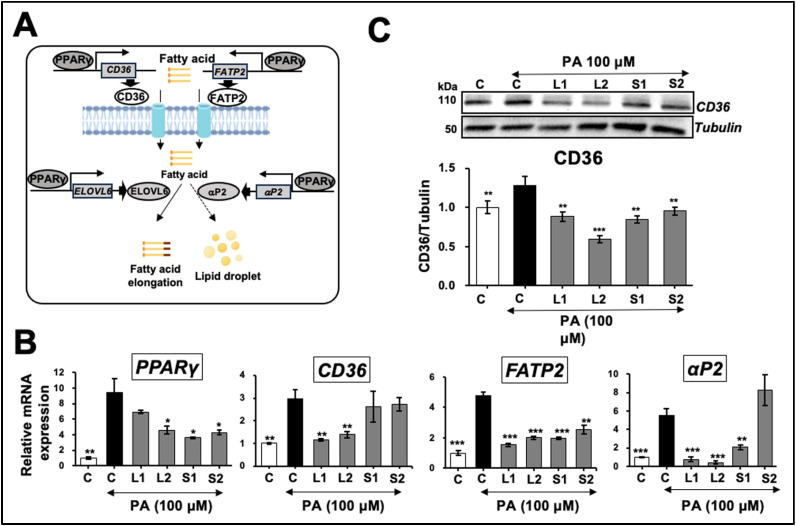
Effects of Ashwagandha extracts on fatty acid uptake and lipogenesis pathway. (**A**) Schematic diagram of fatty acid uptake and lipogenesis controlled by peroxisome proliferator-activated receptor (PPAR)γ, highlighting the key enzymes and transport proteins involved in these metabolic pathways. (**B**) Ashwagandha extracts decreased PPARγ mRNA and its target genes cluster of differentiation 36 (*CD36*), fatty acid transport protein 2 (*FATP2*), and fatty-acid-binding protein 4 (*αP2*) in palmitic acid (PA)-treated Huh7 cells. (**C**) Western blot showing a decrease in CD36 protein in Huh7 cells treated with PA and Ashwagandha extracts compared with PA alone. Quantitation from three independent experiments is shown below. For the quantitation, data were normalized against the PA group and plotted as fold differences (mean ± SD; *n* = 3). The statistical significance of the data (mean ± SD; *n* = 3; and * *p* < 0.05, ** *p* < 0.01, and *** *p* < 0.001) was calculated using an unpaired Student’s *t*-test.

Lipid uptake and lipogenesis: We next investigated the effect of our defined extracts on lipid uptake. FFAs are internalized into the cell cytoplasm by plasma-membrane-associated proteins, including FA translocase CD36, a transcriptional target of PPARγ [44], and fatty acid transport protein (FATP). The latter facilitates the transport of hydrophobic fatty acids to different subcellular compartments [45]. After being translocated into hepatocytes, the FFAs undergo TG synthesis and are stored in lipid droplets; another PPARγ target gene, fatty acid-binding protein (αP2), catalyzes the process [46] (Figure 6A). The expression of *PPARγ* mRNA and its target genes *CD36*, *FATP2*, and *αP2* significantly increased with the PA treatment compared with the control cells (Figure 6B). The *PPARγ* expression decreased in the L1-, L2-, S1-, and S2-pretreated groups. *CD36* decreased in the L1- and L2-pretreated groups, *FATP2* decreased in all four groups, and the *αP2* expression decreased in the L1-, L2-, and S1-treated cells (Figure 6B). Western blotting endorsed these results, as shown in Figure 6C; CD36 protein was induced by the PA treatment and showed a decrease in the cells pretreated with extracts. These results suggest that the Ashwagandha extracts caused a decrease in lipid uptake and lipogenesis, yielding a downregulation of lipid accumulation.

**Figure 7:**
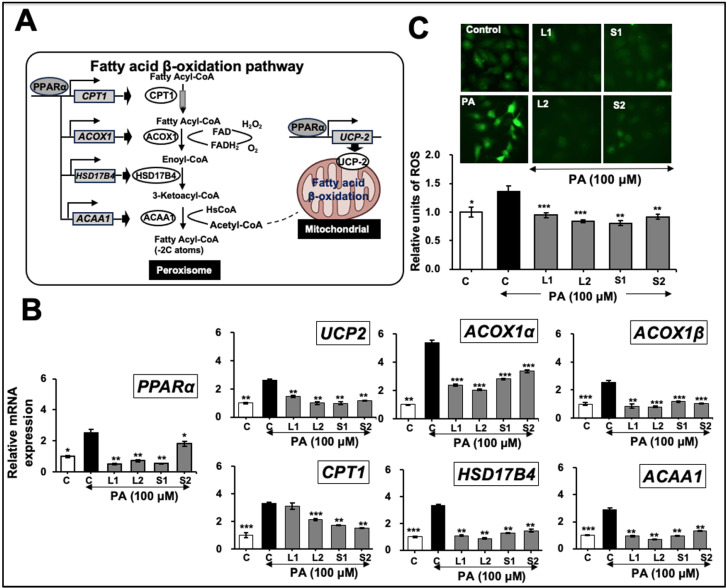
Effects of Ashwagandha extracts on fatty acid β-oxidation and reactive oxygen species (ROS) accumulation. (**A**) Schematic representation of the fatty acid β-oxidation pathway, highlighting key enzymes and regulatory proteins involved in this metabolic process. (**B**) Ashwagandha extracts decreased peroxisome proliferator-activated receptor (*PPAR*)α mRNA and its target genes acyl-CoA oxidase 1 (*ACOX1*), carnitine palmitoyltransferase 1 (*CPT1*), 17β-hydroxysteroid dehydrogenase type IV (*HSD17B4*), and acetyl-coenzyme A acyltransferase 1 (*ACAA1*) in Huh7 cells treated with both PA and Ashwagandha extracts compared with the PA treatment alone. (**C**) Cells treated with PA showed an increase in ROS. Ashwagandha extracts decreased the PA-induced ROS level. Quantitation from three independent experiments is shown below. For the quantitation graphs, data were normalized against the PA group and plotted as fold differences (mean ± SD; *n* = 3). The statistical significance of the data (mean ± SD; *n* = 3; and * *p* < 0.05, ** *p* < 0.01, and *** *p* < 0.001) was calculated using an unpaired Student’s *t*-test. The images in **C** were captured under a magnification of 20×.

Hepatic lipid β-oxidation: long-chain fatty acids preferably undergo oxidation in peroxisome for peroxisomal β-oxidation. PPARα is a nutritional sensor of the rate of fatty acid catabolism. It is a transcription factor that regulates the genes involved in peroxisomal and mitochondrial β-oxidation [24,47]. Very-long-chain fatty acids are preferably oxidized in the peroxisome. As shown in Figure 7A, fatty acids undergo successive rounds of 2-carbon chain-shortening within the peroxisome. The process involves the enzymatic activities of acyl-CoA oxidase 1 (ACOX1), 17β-hydroxysteroid dehydrogenase type IV (HSD17B4), and acetyl-coenzyme A acyltransferase 1 (ACAA1) [47,48]. Mitochondrial β-oxidation is the primary route for the oxidation of most FAs found in hepatocytes. Fatty acids enter the mitochondria through carnitine palmitoyltransferase 1 (CPT1) in the outer mitochondrial membrane [48].

In PA-treated cells, the mRNA expression of *PPARα* and its downstream effector genes, uncoupling Protein 2 (*UCP2*), *ACOX1α*, *ACOX1β, CPT1*, *HSD17B4*, and *ACAA1* involved in the fatty acid β-oxidation pathway, showed an increase compared with the control (Figure 7B). Cells subjected to pretreatments with Ashwagandha extracts L1, L2, S1, and S2 showed a decreased expression of *PPARα*. The expression of six downstream effectors of *PPARα* (*UCP2*, *ACOX1α*, *ACOX1β*, *CPT1*, *HSD17B*, and *ACAA1*) was also examined. As shown in Figure 7B, all genes were downregulated in all four treatment groups (L1, L2, S1, and S2) compared with the control. As ROS levels are considerably upregulated during peroxisomal and mitochondrial β-fatty acid oxidation [49], we next performed a ROS assay for the control and treatment groups. As shown in Figure 7C, the increase in ROS level from the PA treatment was reversed in cells treated with Ashwagandha extracts L1, L2, S1, and S2.

## 3. Discussion

It has recently been demonstrated that CARF (CDKN2AIP) expression is significantly suppressed under free fatty acid-induced metabolic stress [32,33]. A reduction in CARF led to increased ectopic fat accumulation in liver cells, contributing to the development of hepatic steatosis. Notably, the study showed that the overexpression of CARF in HepG2 cells effectively reduced hepatic fat accumulation, endorsing a protective role of CARF against fatty liver disease [33]. Our study further strengthens the critical role of CARF in managing hepatic lipid homeostasis and its downregulation under metabolic stress conditions (Supplementary Figure S1). Furthermore, using the CARF expression as an assay system, we could demonstrate the effect of Ashwagandha-derived withanolides (steroidal lactones with 22-hydroxy-ergostan-26-oic acid-26 and 22-lactone as a withanolide skeleton) on fatty-acid-induced metabolic stress in hepatocytes in vitro. We found that most of the withanolides and the extracts from the leaf and stem of Ashwagandha used in this study caused a reversal of the downregulation of lipid-stress-induced CARF. Of note, the effect was endorsed by reduced ROS levels, protein aggregation, hypoxia, and mitochondria stress (Figure 2 and Figure 3). The oxidative-stress-induced increase in DNA damage (γH_2_AX foci) was also attenuated in treated cells (Figure 2D). Notably, two withanolides, Wi-V and Wi-12dO, enhanced the oxidative-stress-induced increase in ROS and γH2AX foci, suggesting specific activities of different withanolides. Wi-A and Wi-N have the same chemical formula and molecular weight (C_28_H_38_O_6_; 470.6 g/mol). Wi-A is a C5, C6 epoxy compound with hydroxyl groups on C4 and C27, while Wi-N is a C_6_, C_7_ epoxy compound with hydroxyl groups on C_5_ and C_17._ Differential activities of these two withanolides have been reported earlier [3,7,8,19,42]. In the current study, low non-toxic concentrations of Wi-A and Wi-N for hepatocytes in in vitro lipid accumulation stress models exhibited a more substantial effect of Wi-A compared with Wi-N, which was further endorsed by experiments using leaf (rich in Wi-A) and stem (rich in Wi-N) extracts (Figure 4).

Molecular analyses using Ashwagandha extracts from leaf or stem extracts enriched with either Wi-A or Wi-N suggested that the protection of hepatocytes from PA-induced lipid accumulation was driven by the downregulation of PPARγ-induced free fatty acid uptake and hepatic lipogenesis. Furthermore, we found that protection against PA-induced lipid accumulation stress was marked by a decreased mRNA expression of the lipogenesis-related genes *SREBP-1c*, *ACC1*, *FASN*, *SCD1*, and *PPARγ*. The results were supported by an earlier in vivo study of HFD-diet-fed mice. It was demonstrated that consumption of Wi-A (1.25 mg/kg/day) effectively reduced the mRNA expression of the regulators of lipid metabolism (*PPARγ*, *PPARα*, *CD36*, *FASN*, and *CPT1*) [13]. PPARγ is a master regulator of the genes involved in lipid metabolism, free fatty acid import, and TG generation. It upregulates the expression of *CD36* and *αP2* [24,50,51]. Notably, the high expression of *CD36* in hepatocytes has been related to hepatic inflammation and fibrogenesis, which are closely correlated with hepatic diseases [52]. αP2 is also a therapeutic target for diabetes [53]. As endorsed by the mRNA analysis, the four types of Ashwagandha extracts used in this study effectively reduced the mRNA and protein expression of *PPARγ* and its downstream effectors.

An increase in de novo lipogenesis has been closely linked to the pathogenesis of NAFLD [43,54]. SREBP-1c, a key regulator of de novo lipogenesis and its downstream targets ACC1, FASN, and SCD1, is transcriptionally regulated by various nutritional and hormonal factors, including insulin and AMPK [43,55]. We found that the expression of *SREBP-1c* mRNA, *ACC1*, *FASN*, and *SCD1* was downregulated in cells subjected to treatments with Ashwagandha extracts before PA stress (Figure 5B). A protein analysis showed an increase in the precursor but not the mature form of SREBP-1c in cells subjected to PA treatment. Of note, Ashwagandha-extract-treated cells showed a decrease in the PA-induced increase in the SREBP-1c precursor form and matched the changes in mRNA.

De novo lipogenesis and fatty acid oxidation are closely linked. Although the latter is an adaptive response to release lipid accumulation, it generates considerable ROS that promote oxidative stress, inflammation, and hepatic dysfunction [56]. NAFLD is characterized by a dramatic increase in hepatic fatty acid uptake, de novo lipogenesis, and the compensatory enhancement of fatty acid oxidation that promotes cellular damage and disease progression [57]. As shown in Figure 7, PA treatment caused an increase in the mRNA expression of the genes involved in β-oxidation (*PPARα*, *UCP2*, *ACOX1α*, *ACOX1β*, *CPT1*, *HSD17B4*, and *ACAA1*). Notably, cells treated with Ashwagandha extracts showed a decreased PA-induced expression of these genes. In an HFD-fed mice-model study, Wi-A supplementation was shown to improve the hepatic oxidative functions of obese mice by augmented antioxidant enzyme activities [13].

Furthermore, non-toxic low doses of both Wi-A and Wi-N have been reported to offer a variety of bioactivities, including antioxidative, anti-inflammatory, and anti-ferroptosis [3,13,58,59,60,61,62,63,64,65], and, hence, are suggested to enhance normal liver function. Taken together with the current results on protecting hepatocytes from lipid accumulation stress, low non-toxic concentrations of Ashwagandha leaf and stem extracts (possessing Wi-A and Wi-N) are suggested to be useful natural drugs for the management of steatosis and NAFLD.

## 4. Materials and Methods

### 4.1. Cell Culture

HepG2 (American Type Culture Collection, Manassas, VA, USA) and Huh7 (Japanese Collection of Research Bioresources, Tokyo, Japan) cells were cultured in Dulbecco’s modified eagle medium (DMEM, Fujifilm Wako, Osaka, Japan) supplemented with 10% fetal bovine serum and a 1% penicillin–streptomycin–amphotericin B suspension (X100) in a humidified incubator containing 5% CO_2_ at 37 °C. All treatments and assays were performed using ~70–80%-confluent cultures.

### 4.2. Drugs and Treatment

Sodium palmitate acid (PA; Sigma–Aldrich, St. Louis, MO, USA) was dissolved in methanol in a water bath set at 55 °C for 1 h to create 100 mM PA stock and diluted to 5 mM with warm 10% BSA. A stock solution of 5 mM PA was diluted in DMEM to produce the required concentration for the cell treatment. Oleic acid (OA; Fujifilm Wako, Osaka, Japan) and PA (Fujifilm Wako, Osaka, Japan) were mixed at a ratio of 2:1 to configure the FFAs. Purified withanolides (Withaferin A (Wi-A), Withanone (Wi-N), Withanolide B (Wi-B), Withanoside IV (Wi-IV), Withanoside V (Wi-V), Withanostraminolide-12 deoxy (Wi-12dO), Methoxy-withaferin A (mWi-A), and Withanolide A (Wid-A)) were procured from Fuji Film, Japan. The four types of Ashwagandha leaf (L) and stem (S) extracts with (i) a high amount of total withanolides (a high ratio of Wi-A (L1) and a high ratio of Wi-N (L2)) and (ii) a low amount of total withanolides (a high ratio of Wi-A (S1) and a high ratio of Wi-N (S2)) were prepared by methods described earlier [42]. Cells were treated with non-toxic concentrations of purified compounds and extracts, as determined by an MTT-based cell viability assay.

### 4.3. Cytotoxicity Assay

The cells (5 × 10^3^/well) were seeded in a 96-well plate (TPP, Trasadingen, Switzerland) and allowed to adhere overnight by incubating them at 37 °C in a humidified CO_2_ incubator. The cytotoxicity of the FFAs (0.125, 0.25, 0.5, and 1 mM), PA (100, 200, and 400 μM), H_2_O_2_ (25, 50, 100, 200, and 400 μM), NaAsO_2_ (1, 2, 5, 10, 20, and 40 μM), and CoCl_2_ (10, 20, 50, 100, and 200 μM) was determined by an MTT (3-(4,5-dimethylthiazol-2-yl)-2,5-diphenyltetrazolium bromide; Sigma-Aldrich, Tokyo, Japan)-based cell viability assay using the standard method recommended by the manufacturer. Non-toxic concentrations of FFAs and PA were used to treat cells to induce FFA accumulation, followed by their recovery in normal or test reagents (purified withanolides or Ashwagandha extracts) in a supplemented medium. Stressed cells that showed a ~20–30% reduction in viability were subjected to recovery in a medium supplemented with Ashwagandha extracts as indicated. The luminescence was recorded using a 96-well-plate-reading luminometer (TECAN Infinite 200Pro, Melbourne, VIC, Australia).

### 4.4. Oil Red O Staining

The cells (1 × 10^5^) were seeded in 12-well culture dishes (TPP, Trasadingen, Switzerland) and allowed to adhere overnight by incubating them at 37 °C in a humidified CO_2_ incubator. Control and treated (as indicated in the figure legends) cells were fixed with 4% PFA for 1 h at 4 °C, followed by washing with PBS twice and a pretreatment in 80% propylene glycol (Wako, Japan) for 5 min. The cells were then incubated with the Oil Red O solution (0.5% in propylene glycol) (Sigma–Aldrich, Tokyo, Japan) for 1 h at room temperature. The stained cells were rinsed with 80% propylene glycol twice and washed with PBS twice for 5 min each. The cells were photographed using a microscope (ECLIPSE TE300, Nikon, Tokyo, Japan). The lipid content was quantified using ImageJ (National Institute of Health, Bethesda, MD, USA). The lipid particles were also solubilized using 85% isopropanol (Wako, Japan) for 15 min, and the absorbance was measured at 510 nm using a microplate-reading luminometer (TECAN Infinite 200Pro, Melbourne, VIC, Australia).

### 4.5. LipiRed Assay

The cells (6 × 10^4^/well) were seeded on 18 mm glass coverslips, placed in 12-well plates, and adhered overnight. Control and treated cells were washed with PBS twice and treated with 1 μM of a LipiRed (Dojindo Molecular Technologies, Kumamoto, Japan) DMSO solution in phenol-free EMEM medium. They were incubated at 37 °C in a humidified CO_2_ incubator for 30 min. The images were visualized using a Carl Zeiss microscope (Axiovert 200M, Tokyo, Japan).

### 4.6. Triglyceride (TG) Assay

The cells (4 × 10^5^/well) were seeded in 6 cm culture dishes (TPP, Trasadingen, Switzerland) and adhered overnight. Control and treated cells were collected after the experimental treatment, as mentioned above. The total TG levels were detected using a Triglyceride-Glo assay kit (Promega, Madison, WI, USA), following the manufacturer’s instructions. The luminescence was recorded using a 96-well-plate-reading luminometer (TECAN Infinite 200Pro, TECAN, Melbourne, VIC, Australia).

### 4.7. Western Blotting

The cells (4 × 10^5^/well) were seeded in 6 cm culture dishes. After overnight incubation, the cells were treated as indicated, followed by their collection by trypsinization and lysis by shaking (30 min at 4 °C) in a RIPA Lysis Buffer (Thermo Fisher Scientific, Waltham, MA, USA) containing a complete protease inhibitor cocktail (Roche Applied Science, Mannheim, Germany). The lysates were centrifuged at 15,000 rpm for 15 min at 4 °C. The protein concentration of the supernatants (whole-cell lysates) was measured using a Pierce BCA Protein Assay Kit (Thermo Fisher Scientific, Waltham, MA, USA). The cell lysates (20 μg) were separated using 8% SDS–polyacrylamide gel electrophoresis (SDS-PAGE) and transferred to a polyvinyl dene difluoride (PVDF) membrane (Millipore, Billerica, MA, USA) using a semidry transfer blotter (ATTO Corporation, Tokyo, Japan). The membranes were blocked with 3% bovine serum albumin (BSA) at room temperature for 1 h. The blocked membranes were probed with the following target-protein-specific primary antibodies at 4 °C overnight: CARF (raised in our lab [66]), SREBP-1c (sc-13551, Santa Cruz Biotechnology, Paso Robles, CA, USA), fatty acid synthase (FASN) (NBP1-84733, Novus biologicals, Littleton, CO, USA), PPARγ (PA50194884, Thermo Fisher Scientific, Waltham, MA, USA), and CD36 (ab252922, Abcam, Tokyo, Japan). The blots were incubated first with antibodies at room temperature for 1 h, followed by washings in TBST (Tris-buffered saline with 0.1% Tween 20). The following corresponding secondary antibodies were conjugated to horseradish peroxidase: anti-rabbit IgG (31460, Thermo Fisher Scientific, Waltham, MA, USA) or anti-mouse IgG (31430, Thermo Fisher Scientific, Waltham, MA, USA). The antibody-reactive protein bands were detected by an enhanced chemiluminescence reaction (ECL) (GE Healthcare, Amersham, Buckinghamshire, UK). The β-actin antibody (643807, BioLegend, Tokyo, Japan) and α-tubulin (T5168, sigma-Aldrich, St. Louis, MO, USA) were used to detect β-actin or α-tubulin as an internal loading control. Quantitation of the protein expression was determined using ImageJ software (Java 13.0.6 (64-bit), National Institute of Health, Bethesda, MD, USA).

### 4.8. Immunofluorescence

HepG2 cells were harvested and seeded (3 × 10^4^/well) on 18 mm glass coverslips in a 12-well plate for 24 h. After treatment with FFAs (1 mM) for 24 h or treatment to evaluate the effects of Ashwagandha extracts on lipid accumulation following stress with H_2_O_2_ (400 μM) or CoCl_2_ (100 μM) as described above, HepG2 cells were fixed with a mixture of methanol/acetone (1:1, *v*/*v*) for 10 min at 4 °C. After this, they were rinsed three times with PBS and then washed with PBS-Triton X-100 (PBST) for 15 min. A 2% BSA solution was prepared using PBST and blocked at room temperature for 1 h. The coverslips were incubated with specific primary antibodies, including CARF (raised in our lab), γH2AX (9718s, Cell Signaling Technologies, Danvers, MA, USA), and HIF-1α (NB100-479, Novus Biologicals, Littleton, CO, USA) at 4 °C overnight. Secondary antibodies conjugated with Alexa-488 or Alexa-594 (Molecular Probes, Thermo Fisher Scientific, Tokyo, Japan) were added to the plates for a 1 h incubation. Finally, nuclei were stained using Hoechst 33342 (Thermo Fisher Scientific, Tokyo, Japan). The coverslips were fixed on slides and photographed for observation using a microscope (Axiovert 200M, Carl Zeiss, Tokyo, Japan). Finally, the protein expression content was quantified using ImageJ (Java 13.0.6 (64-bit), National Institute of Health, Bethesda, MD, USA).

### 4.9. Quantitative Real-Time PCR

The total RNA from HepG2 or Huh7 cells was extracted using an RNeasy mini kit (Qiagen, Stanford Valencia, CA, USA). RNA was reverse-transcribed using a QuantiTect reverse transcription kit (Qiagen, Tokyo, Japan). Real-time PCR (polymerase chain reaction) was performed using the SYBR Select Master Mix’s method (Applied Biosystem, Life Technologies, Foster City, CA, USA). RT-qPCR was conducted using a real-time system (illumine. Inc.). The PCR amplification reactions consisted of an initial 10 min denaturation step at 95 ℃, followed by 40 cycles at 95 °C for 15 s, 60 °C for 1 min, 72 °C for 45 s, and a final 10 min annealing step at 72 °C. The geometric mean of housekeeping gene 18S was used as an internal control to normalize the variability in the expression levels. The sequences of primers used are given in Supplementary Table S1.

### 4.10. Reactive Oxygen Species (ROS) Assay

A ROS assay was carried out using an Image-ITTM LIVE Green Reactive Oxygen Species Detection Kit (Molecular Probes, Thermo Fisher Scientific, Tokyo, Japan), following the manufacturer’s instructions. Cells (1 × 10^5^/well) were seeded in 12-well culture dishes and adhered overnight. The cells were treated with a culture medium/solvent and H_2_O_2_ (400 μM) or PA (100 μM) were used as untreated, oxidative stress and lipid stress controls. Tert-butyl hydroperoxide (TBHP) provided in the kit was taken as a positive control for ROS induction. Images were captured using a Carl Zeiss microscope (Axiovert 200M, Tokyo, Japan) and analyzed using AxioVision 4.6 software (Carl Zeiss, Tokyo, Japan). The ROS fluorescence intensity was calculated using Image J software (Java 13.0.6 (64-bit), National Institute of Health, Bethesda, MD, USA).

### 4.11. Protein Aggregation Assay

The cells (1.5 × 10^5^ cells/well) were inoculated in 12-well plates and cultured overnight, followed by the transfection of a plasmid-expressing green fluorescent protein (GFP) using X-tremeGENE HP DNA Transfection Reagent (Roche, Tokyo, Japan). Cells were treated with NaAsO_2_ (40 μM) for 24 h post-transfection and recovered in a medium supplemented with Ashwagandha extract for 48 h. The cells were then observed, photographed using a Carl Zeiss microscope (Axiovert 200M, Tokyo, Japan), and quantitated using Image J software (Java 13.0.6 (64-bit), National Institute of Health, Bethesda, MA, USA).

### 4.12. Mitochondrial Membrane Potential (ΔΨm) Assay

The mitochondrial membrane potential of the control and treated cells was detected using JC-1 staining dye (ab141387, Abcam). Briefly, the cells (3 × 10^4^/well) were plated on 18 mm glass coverslips, placed in 12-well plates, and treated with H_2_O_2_ (400 μM) for 24 h, followed by the Ashwagandha extracts described above. Control and treated cells were stained with JC-1 dye (10 μg/mL) in a CO_2_ incubator at 37 °C for 30 min and washed with phosphate-buffered saline (PBS). Green (JC-1 monomers) and red (JC-1 aggregates) fluorescence was observed using a Carl Zeiss microscope (Axiovert 200M, Tokyo, Japan) and analyzed using Image J software (Java 13.0.6 (64-bit), National Institute of Health, Bethesda, MA, USA).

### 4.13. Statistical Analysis

Data were normalized against the control group and expressed as the mean ± standard deviation (SD) value of at least three experimental sets. The statistical significance was calculated using an unpaired Student’s *t*-test and GraphPad software (Prism 10 for macOS, version 10.0.0), and shown as ^ns^ *p* ≥ 0.05 (statistically non-significant), * *p* < 0.05 (statistically significant), ** *p* < 0.01 (statistically very significant), and *** *p* < 0.001 (statistically highly significant).

## 5. Conclusions

Ashwagandha leaf and stem extracts possessing Wi-A and Wi-N have the potential to protect hepatocytes from lipid accumulation stress, steatosis, and NAFLD in vitro, and warrant further laboratory and clinical studies.

## Data Availability

All datasets used and/or analyzed during the current study are available in the manuscript and Supplementary Information files.

## Supplementary Materials

The following supporting information can be downloaded at: https://www.mdpi.com/article/10.3390/ijms252212256/s1.

## List of Abbreviations

ACAA1: Acetyl-CoA acyltransferase; ACC1: Acetyl-CoA Carboxylase 1; ACOX1: acyl-CoA oxidase 1; αP2: fatty-acid-binding protein 4 (FABP4); CARF: collaborator of ARF (CDKN2AIP); CD36: cluster of differentiation 36, also known as fatty acid translocase (FAT); CoCl_2_: cobalt(II) chloride; CPT1: carnitine palmitoyltransferase 1; ER: endoplasmic reticulum; FASN: fatty acid synthase; FATP2: fatty acid transport protein 2; FFAs: free fatty acids; HFD: high-fat diet; HSD17B4: 17β-hydroxysteroid dehydrogenase type IV; IL: interleukin; JC-1: a cationic dye used for mitochondrial membrane potential detection; L1: Ashwagandha leaf extracts with a high amount of total withanolides and a high ratio of Wi-A; L2: Ashwagandha leaf extracts with a high amount of total withanolides and a high ratio of Wi-N; mWi-A: Methoxy-Withaferin A; NAFLD: non-alcoholic fatty liver disease; NASH: non-alcoholic steatohepatitis; PA: palmitic acid; PPARα/γ: peroxisome proliferator-activated receptor alpha/gamma; ROS: reactive oxygen species; S1: Ashwagandha stem extract with a low amount of total withanolides and a high ratio of Wi-A; S2: Ashwagandha stem extract with a low amount of total withanolides and a high ratio of Wi-N; SCD1: stearoyl-CoA desaturase 1; SREBP-1c: sterol regulatory element-binding protein-1c; TG: Triglyceride; TLR4: toll-like receptor 4; TNF-α: tumor necrosis factor alpha; UCP2: uncoupling protein 2; Wi-A: Withaferin A; Wi-B: Withanolide B; Wi-N: Withanone; Wi-IV: Withanoside IV; Wi-V: Withanoside V.

## References

[1] Devabattula G., Panda B., Yadav R., Godugu C. (2024). The Potential Pharmacological Effects of Natural Product Withaferin A in Cancer: Opportunities and Challenges for Clinical Translation. Planta Med..

[2] Kashyap V.K., Peasah-Darkwah G., Dhasmana A., Jaggi M., Yallapu M.M., Chauhan S.C. (2022). *Withania somnifera*: Progress towards a Pharmaceutical Agent for Immunomodulation and Cancer Therapeutics. Pharmaceutics.

[3] Kaul S.C., Wadhwa R. (2017). Science of Ashwagandha: Preventive and Therapeutic Potentials.

[4] Cavaleri F., Chattopadhyay S., Palsule V., Kar P.K., Chatterjee R. (2024). Study of Drug Targets Associated With Oncogenesis and Cancer Cell Survival and the Therapeutic Activity of Engineered Ashwagandha Extract Having Differential Withanolide Constitutions. Integr. Cancer Ther..

[5] Singh N., Yadav S.S., Rao A.S., Nandal A., Kumar S., Ganaie S.A., Narasihman B. (2021). Review on anticancerous therapeutic potential of *Withania somnifera* (L.) Dunal. J. Ethnopharmacol..

[6] Zhang Q., Yuan Y., Cao S., Kang N., Qiu F. (2024). Withanolides: Promising candidates for cancer therapy. Phytother. Res..

[7] Priyandoko D., Ishii T., Kaul S.C., Wadhwa R. (2011). Ashwagandha leaf derived withanone protects normal human cells against the toxicity of methoxyacetic acid, a major industrial metabolite. PLoS ONE.

[8] Konar A., Shah N., Singh R., Saxena N., Kaul S.C., Wadhwa R., Thakur M.K. (2011). Protective role of Ashwagandha leaf extract and its component withanone on scopolamine-induced changes in the brain and brain-derived cells. PLoS ONE.

[9] Wadhwa R., Konar A., Kaul S.C. (2016). Nootropic potential of Ashwagandha leaves: Beyond traditional root extracts. Neurochem. Int..

[10] Chaudhary A., Kalra R.S., Malik V., Katiyar S.P., Sundar D., Kaul S.C., Wadhwa R. (2019). 2,3-Dihydro-3beta-methoxy Withaferin-A Lacks Anti-Metastasis Potency: Bioinformatics and Experimental Evidences. Sci. Rep..

[11] Murthy M.N., Shyamala B.V. (2024). Ashwagandha- *Withania somnifera* (L.) Dunal as a multipotent neuroprotective remedy for genetically induced motor dysfunction and cellular toxicity in human neurodegenerative disease models of Drosophila. J. Ethnopharmacol..

[12] Raut A.A., Rege N.N., Tadvi F.M., Solanki P.V., Kene K.R., Shirolkar S.G., Pandey S.N., Vaidya R.A., Vaidya A.B. (2012). Exploratory study to evaluate tolerability, safety, and activity of Ashwagandha (*Withania somnifera*) in healthy volunteers. J. Ayurveda Integr. Med..

[13] Abu Bakar M.H., Azmi M.N., Shariff K.A., Tan J.S. (2019). Withaferin A Protects Against High-Fat Diet-Induced Obesity Via Attenuation of Oxidative Stress, Inflammation, and Insulin Resistance. Appl. Biochem. Biotechnol..

[14] Xia Y., Wang P., Yan N., Gonzalez F.J., Yan T. (2021). Withaferin A alleviates fulminant hepatitis by targeting macrophage and NLRP3. Cell Death Dis..

[15] Xia Y., Yan M., Wang P., Hamada K., Yan N., Hao H., Gonzalez F.J., Yan T. (2022). Withaferin A in the Treatment of Liver Diseases: Progress and Pharmacokinetic Insights. Drug Metab. Dispos..

[16] Patel D.P., Yan T., Kim D., Dias H.B., Krausz K.W., Kimura S., Gonzalez F.J. (2019). Withaferin A Improves Nonalcoholic Steatohepatitis in Mice. J. Pharmacol. Exp. Ther..

[17] Tekula S., Khurana A., Anchi P., Godugu C. (2018). Withaferin-A attenuates multiple low doses of Streptozotocin (MLD-STZ) induced type 1 diabetes. Biomed. Pharmacother..

[18] Udayakumar R., Kasthurirengan S., Vasudevan A., Mariashibu T.S., Rayan J.J., Choi C.W., Ganapathi A., Kim S.C. (2010). Antioxidant effect of dietary supplement *Withania somnifera* L. reduce blood glucose levels in alloxan-induced diabetic rats. Plant Foods Hum. Nutr..

[19] Paul S., Chakraborty S., Anand U., Dey S., Nandy S., Ghorai M., Saha S.C., Patil M.T., Kandimalla R., Prockow J. (2021). *Withania somnifera* (L.) Dunal (Ashwagandha): A comprehensive review on ethnopharmacology, pharmacotherapeutics, biomedicinal and toxicological aspects. Biomed. Pharmacother..

[20] Mikulska P., Malinowska M., Ignacyk M., Szustowski P., Nowak J., Pesta K., Szelag M., Szklanny D., Judasz E., Kaczmarek G. (2023). Ashwagandha (*Withania somnifera*)-Current Research on the Health-Promoting Activities: A Narrative Review. Pharmaceutics.

[21] Saha P., Ajgaonkar S., Maniar D., Sahare S., Mehta D., Nair S. (2024). Current insights into transcriptional role(s) for the nutraceutical *Withania somnifera* in inflammation and aging. Front. Nutr..

[22] Fabbrini E., Magkos F. (2015). Hepatic Steatosis as a Marker of Metabolic Dysfunction. Nutrients.

[23] Qiu Y., Gan M., Wang X., Liao T., Chen Q., Lei Y., Chen L., Wang J., Zhao Y., Niu L. (2023). The global perspective on peroxisome proliferator-activated receptor gamma (PPARgamma) in ectopic fat deposition: A review. Int. J. Biol. Macromol..

[24] Monroy-Ramirez H.C., Galicia-Moreno M., Sandoval-Rodriguez A., Meza-Rios A., Santos A., Armendariz-Borunda J. (2021). PPARs as Metabolic Sensors and Therapeutic Targets in Liver Diseases. Int. J. Mol. Sci..

[25] Huang L., Tan L., Lv Z., Chen W., Wu J. (2024). Pharmacology of bioactive compounds from plant extracts for improving non-alcoholic fatty liver disease through endoplasmic reticulum stress modulation: A comprehensive review. Heliyon.

[26] Li S., Guo L. (2024). The role of Sirtuin 2 in liver—An extensive and complex biological process. Life Sci..

[27] Tian C., Huang R., Xiang M. (2024). SIRT1: Harnessing multiple pathways to hinder NAFLD. Pharmacol. Res..

[28] Cheung C.T., Kaul S.C., Wadhwa R. (2010). Molecular bridging of aging and cancer: A CARF link. Ann. N. Y. Acad. Sci..

[29] Cheung C.T., Singh R., Kalra R.S., Kaul S.C., Wadhwa R. (2014). Collaborator of ARF (CARF) regulates proliferative fate of human cells by dose-dependent regulation of DNA damage signaling. J. Biol. Chem..

[30] Kalra R.S., Chaudhary A., Omar A., Cheung C.T., Garg S., Kaul S.C., Wadhwa R. (2020). Stress-induced changes in CARF expression determine cell fate to death, survival, or malignant transformation. Cell Stress Chaperones.

[31] Kalra R.S., Chaudhary A., Yoon A.R., Bhargava P., Omar A., Garg S., Yun C.O., Kaul S.C., Wadhwa R. (2018). CARF enrichment promotes epithelial-mesenchymal transition via Wnt/beta-catenin signaling: Its clinical relevance and potential as a therapeutic target. Oncogenesis.

[32] Hasan K.M., Parveen M., Pena A., Calles E.G., Gergis M., Espinoza-Derout J., Sinha-Hikim A., Friedman T.C. (2021). CARF (CDKN2AIP) Regulates Hepatic Lipid Metabolism and Protects Against Development of Non-Alcoholic Fatty Liver Diseases. J. Endocr. Soc..

[33] Hasan K.M., Parveen M., Pena A., Bautista F., Rivera J.C., Huerta R.R., Martinez E., Espinoza-Derout J., Sinha-Hikim A.P., Friedman T.C. (2023). Fatty Acid Excess Dysregulates CARF to Initiate the Development of Hepatic Steatosis. Cells.

[34] Chaudhary A., Kalra R.S., Huang C., Prakash J., Kaul S.C., Wadhwa R. (2017). 2,3-Dihydro-3beta-methoxy Withaferin-A Protects Normal Cells against Stress: Molecular Evidence of Its Potent Cytoprotective Activity. J. Nat. Prod..

[35] Ramos M.J., Bandiera L., Menolascina F., Fallowfield J.A. (2022). In vitro models for non-alcoholic fatty liver disease: Emerging platforms and their applications. iScience.

[36] Olsavsky K.M., Page J.L., Johnson M.C., Zarbl H., Strom S.C., Omiecinski C.J. (2007). Gene expression profiling and differentiation assessment in primary human hepatocyte cultures, established hepatoma cell lines, and human liver tissues. Toxicol. Appl. Pharmacol..

[37] Green C.J., Parry S.A., Gunn P.J., Ceresa C.D.L., Rosqvist F., Piché M.-E., Hodson L. (2020). Studying non-alcoholic fatty liver disease: The ins and outs of in vivo, ex vivo and in vitro human models. Horm. Mol. Biol. Clin. Investig..

[38] Gunn P.J., Green C.J., Pramfalk C., Hodson L. (2017). In vitro cellular models of human hepatic fatty acid metabolism: Differences between Huh7 and HepG2 cell lines in human and fetal bovine culturing serum. Physiol. Rep..

[39] Chen X., Li L., Liu X., Luo R., Liao G., Li L., Liu J., Cheng J., Lu Y., Chen Y. (2018). Oleic acid protects saturated fatty acid mediated lipotoxicity in hepatocytes and rat of non-alcoholic steatohepatitis. Life Sci..

[40] Ma W., Wu J.H.Y., Wang Q., Lemaitre R.N., Mukamal K.J., Djoussé L., King I.B., Song X., Biggs M.L., Delaney J.A. (2015). Prospective association of fatty acids in the de novo lipogenesis pathway with risk of type 2 diabetes: The Cardiovascular Health Study233. Am. J. Clin. Nutr..

[41] Mei S., Ni H.-M., Manley S., Bockus A., Kassel K.M., Luyendyk J.P., Copple B.L., Ding W.-X. (2011). Differential Roles of Unsaturated and Saturated Fatty Acids on Autophagy and Apoptosis in Hepatocytes. J. Pharmacol. Exp. Ther..

[42] Kaul S.C., Ishida Y., Tamura K., Wada T., Iitsuka T., Garg S., Kim M., Gao R., Nakai S., Okamoto Y. (2016). Novel Methods to Generate Active Ingredients-Enriched Ashwagandha Leaves and Extracts. PLoS ONE.

[43] Ferré P., Phan F., Foufelle F. (2021). SREBP-1c and lipogenesis in the liver: An update1. Biochem. J..

[44] Tontonoz P., Nagy L., Alvarez J.G., Thomazy V.A., Evans R.M. (1998). PPARgamma promotes monocyte/macrophage differentiation and uptake of oxidized LDL. Cell.

[45] Badmus O.O., Hillhouse S.A., Anderson C.D., Hinds T.D., Stec D.E. (2022). Molecular mechanisms of metabolic associated fatty liver disease (MAFLD): Functional analysis of lipid metabolism pathways. Clin. Sci..

[46] Makowski L., Brittingham K.C., Reynolds J.M., Suttles J., Hotamisligil G.S. (2005). The fatty acid-binding protein, aP2, coordinates macrophage cholesterol trafficking and inflammatory activity. Macrophage expression of aP2 impacts peroxisome proliferator-activated receptor gamma and IkappaB kinase activities. J. Biol. Chem..

[47] Tahri-Joutey M., Andreoletti P., Surapureddi S., Nasser B., Cherkaoui-Malki M., Latruffe N. (2021). Mechanisms Mediating the Regulation of Peroxisomal Fatty Acid Beta-Oxidation by PPARα. Int. J. Mol. Sci..

[48] Todisco S., Santarsiero A., Convertini P., De Stefano G., Gilio M., Iacobazzi V., Infantino V. (2022). PPAR Alpha as a Metabolic Modulator of the Liver: Role in the Pathogenesis of Nonalcoholic Steatohepatitis (NASH). Biology.

[49] Luukkonen P.K., Porthan K., Ahlholm N., Rosqvist F., Dufour S., Zhang X.M., Lehtimäki T.E., Seppänen W., Orho-Melander M., Hodson L. (2023). The PNPLA3 I148M variant increases ketogenesis and decreases hepatic de novo lipogenesis and mitochondrial function in humans. Cell Metab..

[50] Daquinag A.C., Gao Z., Fussell C., Immaraj L., Pasqualini R., Arap W., Akimzhanov A.M., Febbraio M., Kolonin M.G. (2021). Fatty acid mobilization from adipose tissue is mediated by CD36 posttranslational modifications and intracellular trafficking. JCI Insight.

[51] Abumrad N.A., Cabodevilla A.G., Samovski D., Pietka T., Basu D., Goldberg I.J. (2021). Endothelial Cell Receptors in Tissue Lipid Uptake and Metabolism. Circ. Res..

[52] Wilson C.G., Tran J.L., Erion D.M., Vera N.B., Febbraio M., Weiss E.J. (2016). Hepatocyte-Specific Disruption of CD36 Attenuates Fatty Liver and Improves Insulin Sensitivity in HFD-Fed Mice. Endocrinology.

[53] Furuhashi M., Tuncman G., Görgün C.Z., Makowski L., Atsumi G., Vaillancourt E., Kono K., Babaev V.R., Fazio S., Linton M.F. (2007). Treatment of diabetes and atherosclerosis by inhibiting fatty-acid-binding protein aP2. Nature.

[54] Donnelly K.L., Smith C.I., Schwarzenberg S.J., Jessurun J., Boldt M.D., Parks E.J. (2005). Sources of fatty acids stored in liver and secreted via lipoproteins in patients with nonalcoholic fatty liver disease. J. Clin. Investig..

[55] Li D., Ikaga R., Yamazaki T. (2018). Soya protein β-conglycinin ameliorates fatty liver and obesity in diet-induced obese mice through the down-regulation of PPARγ. Br. J. Nutr..

[56] Che Z., Zhou Z., Li S.Q., Gao L., Xiao J., Wong N.K. (2023). ROS/RNS as molecular signatures of chronic liver diseases. Trends Mol. Med..

[57] Li Z., Berk M., McIntyre T.M., Gores G.J., Feldstein A.E. (2008). The lysosomal-mitochondrial axis in free fatty acid-induced hepatic lipotoxicity. Hepatology.

[58] Zhang H., Wang J., Prakash J., Zhang Z., Kaul S.C., Wadhwa R. (2023). Three-Way Cell-Based Screening of Antistress Compounds: Identification, Validation, and Relevance to Old-Age-Related Pathologies. J. Gerontol. A Biol. Sci. Med. Sci..

[59] Abeesh P., Guruvayoorappan C. (2023). The Therapeutic Effects of Withaferin A against Cancer: Overview and Updates. Curr. Mol. Med..

[60] Chen X., Zhu N., Wu Y., Zhang Y., Zhang Y., Jin K., Zhou Z., Chen G., Wang J. (2024). Withaferin A, a natural thioredoxin reductase 1 (TrxR1) inhibitor, synergistically enhances the antitumor efficacy of sorafenib through ROS-mediated ER stress and DNA damage in hepatocellular carcinoma cells. Phytomedicine.

[61] Logie E., Vanden Berghe W. (2020). Tackling Chronic Inflammation with Withanolide Phytochemicals-A Withaferin a Perspective. Antioxidants.

[62] Sheng J.D., Liu J., Du J.W., Wang Y.P. (2023). Withaferin A alleviates inflammation and joint injury in arthritic rats via elevating microRNA-1297 to target karyopherin alpha2. J. Physiol. Pharmacol..

[63] Dar N.J., Hamid A., Ahmad M. (2015). Pharmacologic overview of *Withania somnifera*, the Indian Ginseng. Cell Mol. Life Sci..

[64] Zhou Z.X., Cui Q., Zhang Y.M., Yang J.X., Xiang W.J., Tian N., Jiang Y.L., Chen M.L., Yang B., Li Q.H. (2023). Withaferin A inhibits ferroptosis and protects against intracerebral hemorrhage. Neural Regen Res..

[65] Pan Q., Luo Y., Xia Q., He K. (2021). Ferroptosis and Liver Fibrosis. Int. J. Med. Sci..

[66] Hasan M.K., Yaguchi T., Minoda Y., Hirano T., Taira K., Wadhwa R., Kaul S.C. (2004). Alternative reading frame protein (ARF)-independent function of CARF (collaborator of ARF) involves its interactions with p53: Evidence for a novel p53-activation pathway and its negative feedback control. Biochem. J..

