# Supplementary material for: Molecular Insights into the Inhibition of Lipid Accumulation in Hepatocytes by Unique Extracts of Ashwagandha
Source: Int J Mol Sci. 2024 Nov 14;25(22):12256. doi: 10.3390/ijms252212256 (PMC11594306; doi:10.3390/ijms252212256)
Supplement: Supplementary file 1 [file ijms-25-12256-s001.zip › ijms-3248774-supplementary.pdf]

Molecular Insights into the Inhibition of Lipid Accumulation in Hepatocytes by Unique Extracts of Ashwagandha

Dongyang Li[1], Han Hanlin[1,2], Yixin Sun[1,2], Huayue Zhang[1,2], Ren Yoshitomi[1], Sunil C Kaul[1] and Renu Wadhwa[1,2*]

[1] AIST-INDIA DAILAB, National Institute of Advanced Industrial Science and Technology (AIST),
Tsukuba 305-8565, Japan; dongyang.li@technopro.com (D.L.); s2130296@u.tsukuba.ac.jp (H.H.); sun.yixin.36r@st.kyoto-u.ac.jp (Y.S.); s2130297@u.tsukuba.ac.jp (H.Z.); yoshitomi.ren@aist.go.jp (R.Y.);
s-kaul@aist.go.jp (S.C.K.)

[2] Graduate School of Science and Technology, University of Tsukuba, 1-1-1 Tennodai, Tsukuba 305-8577, Japan

* Correspondence: renu-wadhwa@aist.go.jp

Supplementary Figures S1–S3 and Table S1

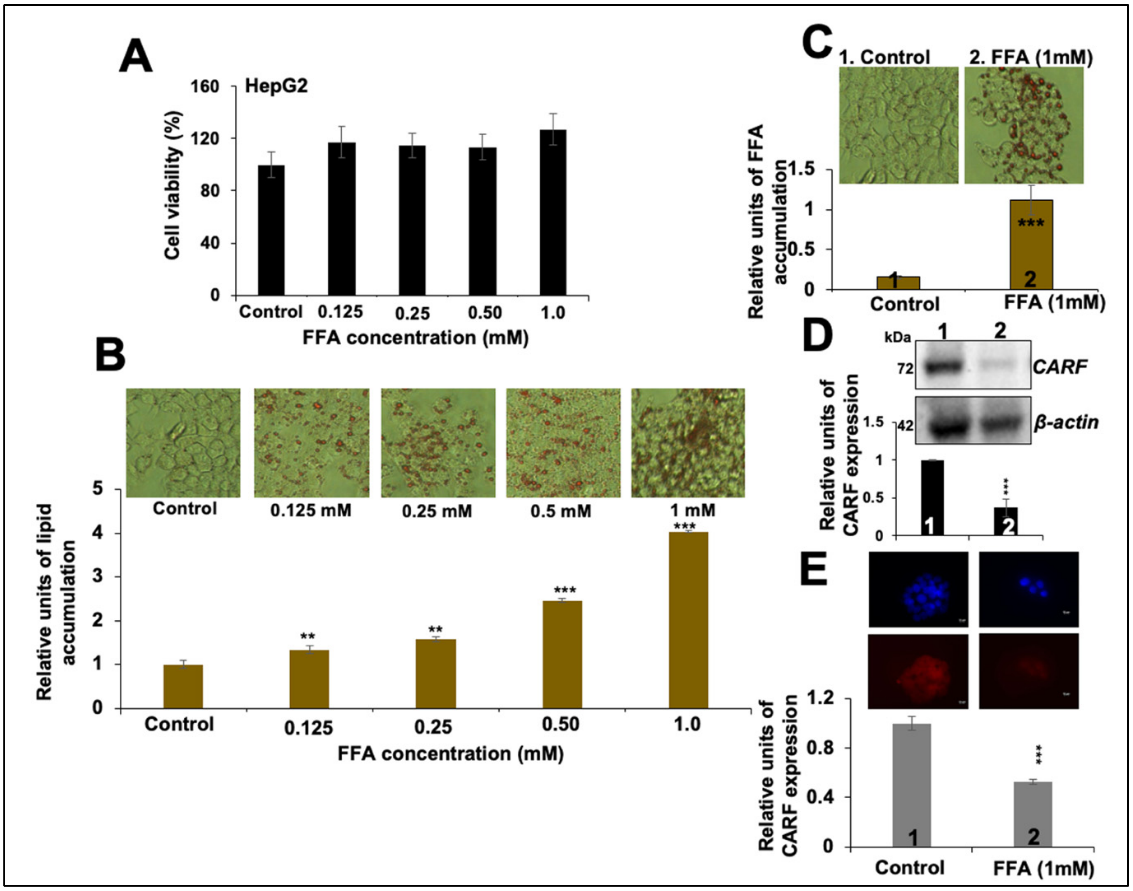

Supplementary Figure S1. FFA treatment caused downregulation of CARF expression. A. Dose-dependent effect of FFA treatment on the viability of HepG2 cells. B. Dose-dependent increase in FFA accumulation in cells as detected by Oil Red O staining. C-E. Decrease in CARF expression in FFA-treated cells as detected by Western blotting and immunostaining. ** $p < 0.01$, and *** $p < 0.001$ denote the statistical significance compared with the control group (derived from an unpaired Student's *t*-test).

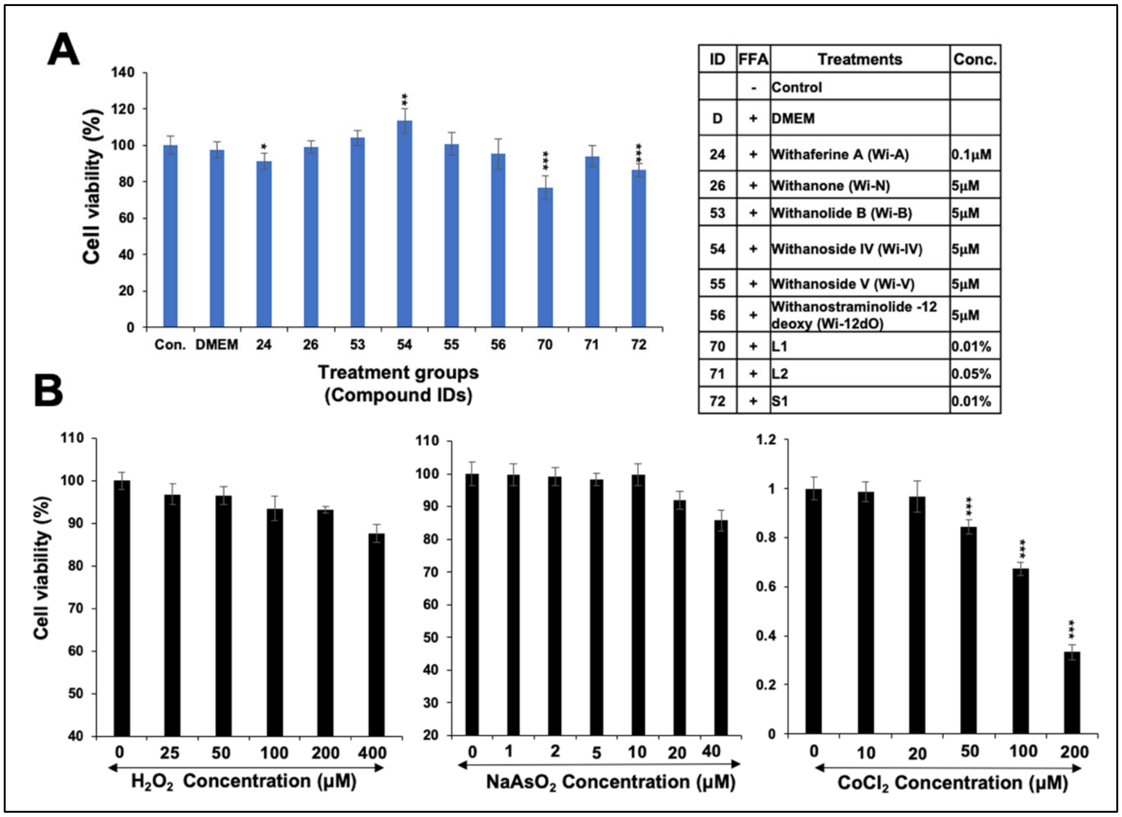

Supplementary Figure S2. Standardization of nontoxic concentrations of withanolides and stress conditions. A. Viability of HepG2 cells treated with Ashwagandha withanolides and extracts (as shown in the Table). B. Dose-dependent effect of H_2O_2, $NaASO_2$, and $CoCl_2$ on viability of HepG2 cells. The statistical significance of the data (mean ± SD; $n = 3$; and * $p < 0.05$, ** $p < 0.01$, and *** $p < 0.001$) was calculated using an unpaired Student's *t*-test.

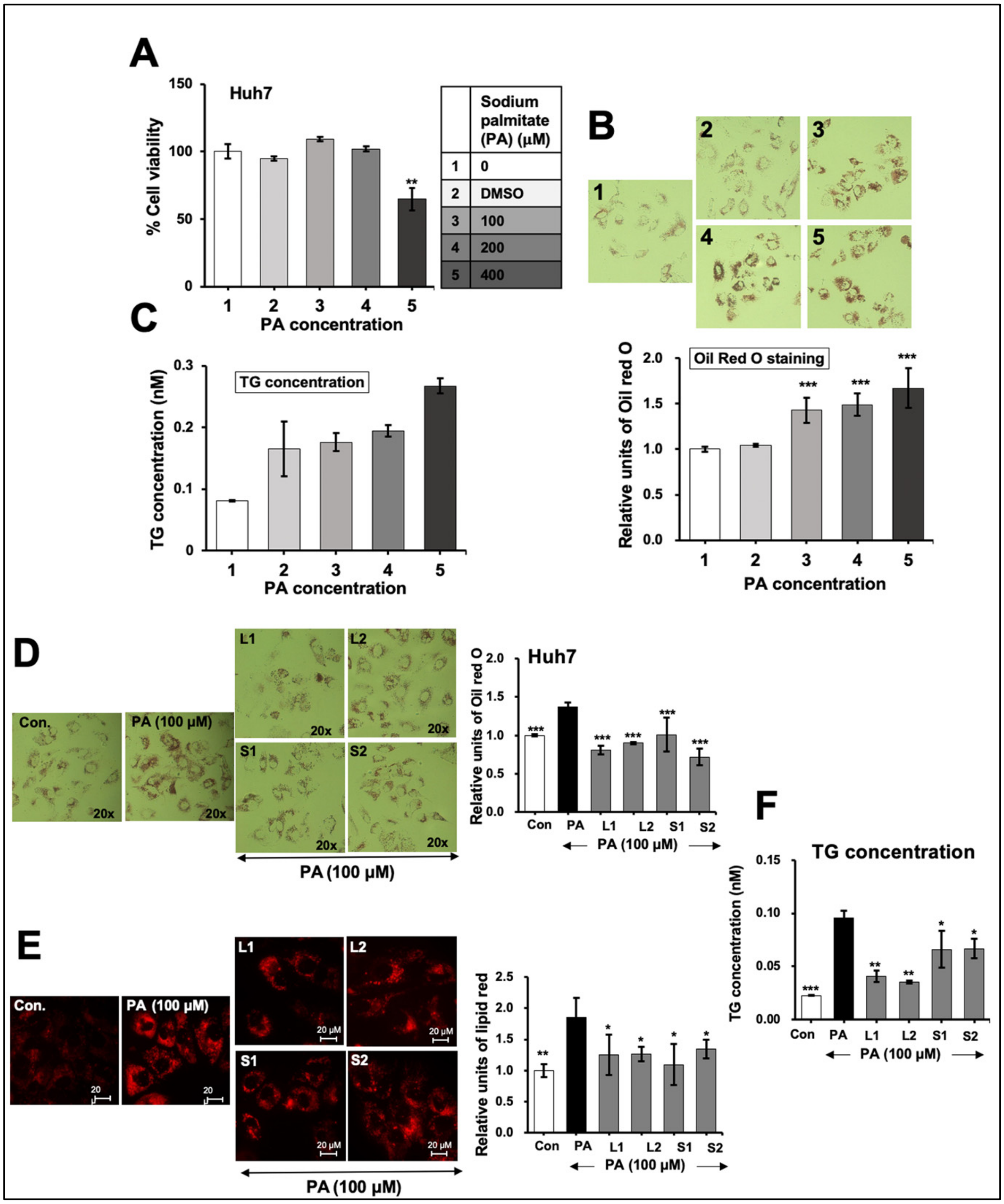

Supplementary Figure S3. Effect of palmitic acid (PA) and Ashwagandha extracts on Huh7 cells. A. Viability of Huh7cells treated with serially increasing concentrations of PA. B and C. Dose-dependent increase in FFA accumulation in Huh7 cells as detected by Oil Red O staining (B) and Triglyceride (TG) assay (C). D and E. Cells treated with a nontoxic concentration of PA showed an increase in FFA accumulation; treatment with Ashwagandha extracts (L1, L2, S1, and S2) caused a decrease as detected by Oil Red O staining and TG assays. The statistical significance of the data (mean ± SD; $n = 3$; and * $p < 0.05$, ** $p < 0.01$, and *** $p < 0.001$) was calculated using an unpaired Student's t-test.

Supplementary Table S1. Sequence of Primers used for quantitative real-time PCR

Primer	Forward 5'-3'	Reverse 5'-3'
CARF	TCAAAGTGACAGATGCTCCA	CGTTGAACTGTTTTCCTGCT
SREBP-1c	GCTGTCCACAAAAGCAAATCTC	GTCAGTGTGTCCTCCACCTCAGT
ACC1	TTCACTCCACCTTGTCAGCGGA	GTCAGAGAAGCAGCCCATCACT
FAS	TTCTACGGCTCCACGCTCTTCC	GAAGAGTCTTCGTCAGCCAGGA
SCD1	CCTGGTTTCACTTGGAGCTGTG	TGTGGTGAAGTTGATGTGCCAGC
PPARγ	TTGAAAGAAGCCAACACTAAACCAC	AATGGCATCTCTGTGTCAACCAT
CD36	TGGAACAGAGGCTGACAACTT	TTGATTTTGATAGATATGGGATGC
FATP2	TACTCTTGCCTTGCGGACTAA	CCGAAGCAGTTCACCGATATAC
αP2	TGCAGCTTCCTTCTCACCTTGA	TCCTGGCCCAGTATGAAGGAAATC
PPARα	ACTTATCCTGTGGTCCCCGG	CCGACAGAAAGGCACTTGTGA
UCP-2	TGTGCCCTTACCATGCTCCA	AGGGCTCGTTTCAGCTGCTC
ACOX1α	TGCTCAGAAAGAGAAATGGC	TGGGTTTCAGGGTCATACG
ACOX1β	CCTCTGGATCTTCACTTGG	TGGGTTTCAGGGTCATACG
CPT1	CTGGACTTCATTCCTGGAAAAAGAAG	CGATCTTGGCGTACATCGTTGTCATC
HSD17B4	ACCAACTCCTTTGAAGTCCCC	GCCCTGGCTTTTGCAGAAA
ACAA1	GAGATCAATGAGGCCTTTGC	CATGGACACCACTCCGTATG
